# Are Nutritional Patterns among Polish Hashimoto Thyroiditis Patients Differentiated Internally and Related to Ailments and Other Diseases?

**DOI:** 10.3390/nu13113675

**Published:** 2021-10-20

**Authors:** Paulina Ihnatowicz, Paweł Wątor, Jerzy Gębski, Joanna Frąckiewicz, Małgorzata Ewa Drywień

**Affiliations:** 1 Department of Human Nutrition, Institute of Human Nutrition Sciences, Warsaw University of Life Sciences (SGGW-WULS), Nowoursynowska 159C, 02-776 Warsaw, Poland; dietetyk.ihnatowicz@gmail.com (P.I.); joanna_frackiewicz@sggw.edu.pl (J.F.); 2 SanDiet Dietetyka & Lifestyle, Dietary Counseling, Pańska 96, 00-837 Warsaw, Poland; wator.dietitian@gmail.com; 3 Department of Food Market and Consumer Research, Institute of Human Nutrition Sciences, Warsaw University of Life Sciences (SGGW-WULS), Nowoursynowska 159C, 02-776 Warsaw, Poland; jerzy_gebski@sggw.edu.pl

**Keywords:** Hashimoto’s disease, pattern analysis, heterogeneity of Hashimoto’s disease, Hashimoto’s patients’ eating patterns

## Abstract

There is not any diet recommended for Hashimoto’s disease, despite that those patients are often undernourished. Because of the high heterogeneity of Hashimoto’s patients, insight into dietary patterns might shed some light on the patient-tailored dietary approach, thus improving their treatment and helping to identify patients with the highest probability of particular nutritional deficiencies. The aim of this study was to identify Hashimoto’s patients’ dietary patterns and their characterization based on both socio-demographic variables and dietary self-assessment. We collected data online from patients with Hashimoto’s disease. The questionnaire formula used in the study was developed based on a validated food frequency questionnaire KomPAN^®^. K-means pattern analyses were used to characterize patients into patterns based on the frequency of particular types of foods consumption and socio-demographic factors. Four patterns were identified. We labeled them as ‘Convenient’, ‘Non-meat’, ‘Pro-healthy’, and ‘Carnivores’ with participants proportions at approximately one-fourth per each pattern. The patients were mainly of the female gender (94.08%), with a female: male ratio of 15.9. Hashimoto’s patients differed in their food product choices, food choice motives, dieting experience, nutritional knowledge, smoking habits, food allergies and intolerances, and lipid disorders, and thus represent different eating patterns. However, these patterns were not determined by comorbidities or the majority of ailments.

## 1. Introduction

Hashimoto’s disease (HT) is one of the most prevalent autoimmune endocrine disorders, causing thyroiditis and hypothyroidism.

A meta-analysis published in 2014 showed that the mean prevalence of undiagnosed hypothyroidism in Europe was 4.94% (4.75–5.13%), the mean prevalence of hypothyroidism was 3.05% (3.01–3.09%), and the incidence rate was 226.2 (222.26–230.17) per 100.000 per year [1]. Another meta-analysis published in 2019, which included more data, showed the prevalence of undiagnosed overt and subclinical hypothyroidism in Europe was on a mean 0.65% and 4.11%, respectively [2]. In 2009, a Polish survey on women’s health provided similar data. There were almost 1.5 million out of 38 million people (3.9%) with thyroid disease diagnoses. In 5 years, from 2004 to 2009, the prevalence of the disease increased by 1.4% [3]. Another study does not support a hypothesis of the growing incidence of autoimmune thyroiditis (AT) and hypothyroidism in Poland. From 2006 to 2013 (8 years), diagnoses of AT and hypothyroidism dropped from 10.4% to 4.8% and from 17.8% to 7.7%, respectively [4].

It is crucial to assess nutritional patterns of HT patients as individual food groups or nutrients are found to have an impact on the risk of HT disease development or progression [5]. What we understand as an unhealthy diet is a high intake of sugar and fat, and a low intake of fruit and vegetables, which was found to be related to an increased risk of various disease development [6]. This unhealthy pattern is similar to the “Traditional Polish” dietary pattern, which is composed of potatoes, sweets, sweetened carbonated beverages, meat, and meat products [7]. Highly refined carbohydrates or fat content in such products were suggested to have the possibility to change the reward neurocircuitry causing ‘food addiction’ and overconsumption [8,9]. This may be one of the barriers for a subject to change dietary behaviors, as it was found that long-term adherence to behavior changes is poor and clinically unsatisfactory [10]. In the study of Krusińska et al., they identified three dietary patterns: ‘Prudent’, ‘Processed and fast food’, and ‘Traditional Polish’ in 320 subjects from northeastern Poland [7]. The latter was composed of food products like potatoes, sweets and sweetened beverages, and meat and meat dishes. Participants belonging to the ‘Prudent’ dietary pattern had a higher consumption of cheese (including curd cheese), fermented milk drinks, fruit, and vegetables, also in the form of juices, wholegrain bread, fish, and milk. The ‘Processed and fast food’ dietary pattern was associated with a higher consumption of ready-to-eat soups, canned food, alcoholic drinks, and fast food [7]. In another study on young polish females, four dietary patterns were found [11], which are similar to Krusińska et al.’s study results [7]. Those patterns are ‘Traditional Polish’ (composed of white bread, potatoes, red meats/fish/eggs, fats, margarine or butter, fried chicken, and wholegrain bread), ‘Fruit and vegetables’ (composed of fruit, vegetables, and beans), ‘Fast-food and sweets’ (composed of hamburger or cheeseburger, ice cream, doughnuts, pastries, cakes or cookies, sweets and snacks, french fries, corn and potato chips or popcorn, and salad dressings or mayonnaise), and ‘Dairy and fats’ (composed of cheese or cheese spread, whole milk, margarine or butter, potatoes, and cereals). Unfortunately, there have not been dietary pattern studies in HT patients. However, based on the current knowledge, we suggest that healthy dietary patterns for HT patients should include a particularly high consumption of vegetables and fruits to decrease deleterious oxidative stress (OS). It was found that OS is associated with HT development, as well as progression from the subclinical to the clinical state of the disease [12]. It would be interesting to see results of the use of dietary patterns like Mediterranean diet [13], Dietary Approaches to Stop Hypertension (DASH) [14], or the paleolithic diet [15] in HT patients as they were found to improve oxidative balance. Vegetarian or lacto-ovo-vegetarian diets are not recommended because studies of their effects yield inconsistent results on glutathione metabolism or OS markers [16]. Those dietary patterns also exclude highly refined carbohydrates, which are known to be a source of oxidative stress [17]. It is also worth noting that a gluten exclusion diet might be beneficial for HT patients; however, it may also exacerbate metabolism [18].

An online questionnaire study among 150 patients with autoimmune thyroid disease showed that 8 out of 10 patients used a lactose or gluten elimination diet. Almost 40% of patients who changed dietary patterns did it based on medical recommendations while 62% did it by themselves. About 80% of participants used the internet as a source for nutrition education for their disease [19]. Studies have shown that Polish HT patients are deficient in various nutrients and have a low-quality diet [20,21]. Moreover, they do not follow medical support in most cases, but those who did were not given medical recommendations that fit guidelines and experts pattern statements [19]. For example, in 2018, a Polish society called POLSPEN stated that a gluten-free diet is justified only in those with celiac disease coexistence [22], while the questionnaire revealed that from 37.3% on gluten-free diet only 2% had celiac disease [19].

There is a need for insight into the heterogeneity of Hashimoto’s patients’ dietary patterns to improve dietary therapy of the condition they suffer from. Based on our own clinical practice, we know that the standard treatment of thyroid dysfunction is thyroid hormone replacement, usually without interference in patients’ nutrition. However, when it is, it is wrong and potentially harmful [19]. Such an approach does not seem to be satisfactory for patients as their quality of life, even after the introduction of levothyroxine therapy, is still decreased despite normal blood thyroid parameters [23]. The quality of life seems to depend on the serum concentration of anti-TPO antibody titers rather than thyroid functions [23,24].

The etiology of HT is not fully explained and understood. However, it is known that disease arises when environmental factors such as nutritional ones occur in genetically susceptible people. The eating patterns of patients with Hashimoto’s disease are not fully recognized. It is known that people with this disease consume red meat, fish, dairy products [25], and animal fats [26] more often than healthy foods. Moreover, they are not likely to change their diet after being diagnosed with HT. We previously published a paper describing our approach to dietary management of Hashimoto’s thyroiditis [5]. In the same year, Wojtas et al. published a graphic-text qualitative dietary protocol (Diet4Hashi) for HT patients [27]. The few studies available showed that higher consumption of animal fats and butter was associated with the risk of developing antibodies to TPOAb and/or TgAb, while a diet rich in vegetables, dried fruit, nuts, and muesli reduced this risk [28]. According to the current findings, the Mediterranean diet pattern is the most beneficial for HT patients, as it has a protective effect due to antioxidant properties [25].

Generally, the common dietary recommendations are less frequently implemented in the pattern of HT patients than in the compared control patterns. There may be multiple reasons for this situation. Undoubtedly, there is still a lack of developed dietary recommendations dedicated to HT patients and they have to rely only on medical specialists’ recommendations or advice found in media. Both sources provide incomplete and often contradictory information [19].

It seems that the design of the dietary recommendations for HT should be individualized and determined on the basis of existing dietary habits, which may differ depending on the country and the specific impact factors existing there like in Poland [29,30,31].

The aim of the study was to identify the eating patterns of patients with HT disease and link them to health problems. We hypothesize that dietary patterns in HT patients differ internally but may not be different from those found in the Polish population generally. Because most of our participants are women with health problems, we would expect that consumption of food products considered healthy (e.g., vegetables) to be higher than those considered as unhealthy (e.g., sweets). 

## 2. Materials and Methods

### 2.1. Study Design and Sample Collection

**Figure 1:**
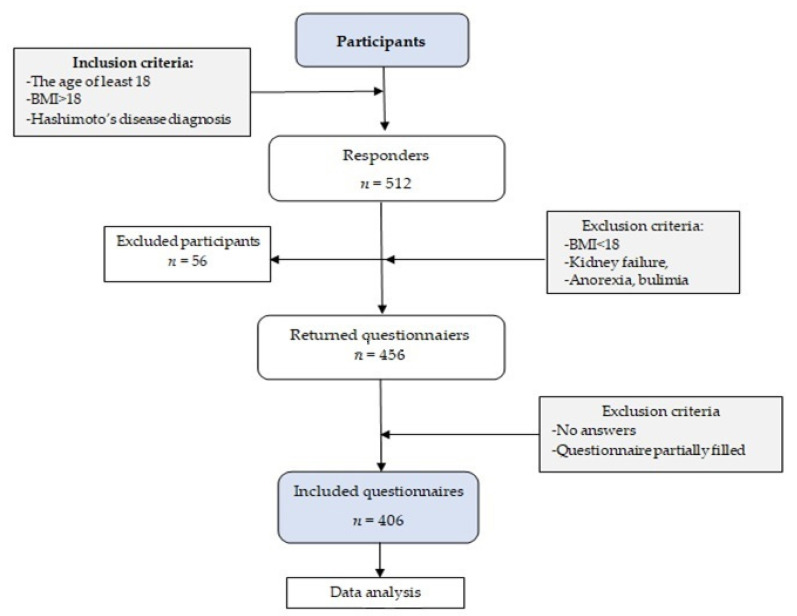
Flowchart: study design and data collection (*n*—number of participants).

This study was performed online using a developed food frequency questionnaire. Study participants were people that voluntarily consented to take a part in the study announced on social media. The questionnaire was directed it specifically to HT patients with diagnosed disease. The questionnaire was developed based on the KomPAN^®^ food frequency questionnaire, which studies dietary habits and nutrition beliefs [32,33]. We checked the frequency of consumption of 34 food products. The exclusion criteria were age under 18, body mass index (BMI) below or equal to 18, anorexia, bulimia, malnutrition, and kidney failure. Inclusion criteria were age and BMI over 18, respectively, and being diagnosed with Hashimoto’s disease. Initially, there were 512 responders that fit the inclusion criteria and 456 left after exclusion. In the final analysis, 406 patients were included as the rest failed to fill the questionnaire (Figure 1).

### 2.2. Eating Habits, Health Status, and Lifestyle 

The variables used for this pattern came from a validated Dietary Habits and Nutrition Beliefs Questionnaire (KomPAN^®^) [32,33]. The variables included the following: eating habits (e.g., number of meals eaten daily, factors affecting food choice decisions, sugar intake, and salt use), HT characteristics and health status (e.g., concurrence of other diseases, HT treatment, symptoms), dieting in previous years and results and thoughts about it, smoking habits (e.g., number of cigarettes smoked daily), sleeping habits (e.g., sleeping time per day daily), physical activity (e.g., work type and number of hours spent watching TV), and nutritional knowledge self-assessment. The data were collected in January 2020 anonymously and entered in a database.

Not all variables collected in the questionnaire were entered and used in the pattern analysis. We selected appropriate data based on the presumption of the clinical relevance of particular variables and expectations to be discriminative in the whole dataset included in the analysis.

### 2.3. Socio-Demographic Variables

Socio-demographic data were collected collectively with other data in the questionnaire. It included gender, age, education, and place of residence. Body mass index (BMI) was calculated based on the self-assessed weight and height and interpreted along with the criteria of the World Health Organization [34]. The ranges BMI 18.5–24.9 kg/m^2^, BMI 25.0–29.9 kg/m^2^, and BMI 30.0 kg/m^2^ were considered normal, overweight, and obese, respectively [35].

### 2.4. Statistical Analysis

The preliminary analysis of the variables characterizing the diet of people with Hashimoto’s disease gave the basis for the use of factor analysis (PCA) with varimax rotation of the factors in order to reduce the analyzed variables and obtain nutritional patterns. The effect of the varimax rotation used was to obtain just such a situation, where one factor has a high value of the load, and the other has a low value. The analysis of the reliability of answers to the question about the frequency of consumption of individual products was carried out using the Cronbach’s alpha coefficient. Its value was 0.873, which confirmed the correct selection of questions to the factor analysis.

For the reduction of variables, factor analysis was used, thanks to which seven factors (dietary patterns) characterizing the consumption of individual group of products were obtained from 34 variables. Each of them represented a group of variables most correlated with each other. The factors included variables whose factor loading was at least 0.4, with the factor adequate for the requirements of factor analysis as studied by the Kaiser–Mayer–Olkin measure (KMO). The Cronbach’s alpha reliability coefficient was used to estimate the reliability of the scale used [36,37]. The Kaiser–Meyer–Olkin (KMO) test was conducted as a measure of how suited the used data is for factor analysis.

The test measures sampling adequacy for each variable in the model and for the complete model. The statistic is a measure of the proportion of variance among variables that might be common variance. The KMO value was 0.862, which confirms the correctness of the application of this method. The total variance explained by factor analysis was 55.08%. The obtained factors (dietary patterns) were normalized by ‘range’ (minimum subtracted and divided by SD) to facilitate their interpretation.

The normalized values of factors were assigned to quintiles, and then clustered using seven obtained nutritional formulas characterizing the consumption of highly processed products, dairy products, meat and its products, other non-recommended foods, vegetables and fruits, fruit and vegetable juices, and water (Table 1).

We used the ANOVA test for factors analyzes with the post hoc Waller–Duncan K-ratio *t*-test to compare the mean values of particular food patterns consumption between pairs of patterns. To assess the relationship between two nominal variables we used the Chi-square test. 

Initial clusterization was carried out with the use of hierarchical methods, which did not give the expected effect. The second one was carried out based on the k-means method using the centers of gravity of patterns derived from the hierarchical method. Thus, four well-separated clusters (patterns) were obtained. The number of patterns was selected based on the dendrogram and statistics: Cubic Patterning Criteria (CCC) and Pseudo F. Moreover, the correct level of pattern differentiation was confirmed by the ANOVA from the post-hoc Waller–Duncan K-ratio *t*-test (Table 2).

Profiling of the selected patterns was carried out with the use of variables describing the socio-demographic characteristics of the respondents, eating habits, etc. For qualitative variables, the χ^2^ test of independence was used, and for quantitative variables, the post-hoc Waller–Duncan K-ratio *t* Test was used.

All statistical analyzes were performed using the SAS 9.4 statistical package at a significance level of *p* < 0.05.

## 3. Results

### 3.1. Sample and Patterns Characteristics 

Pattern 1, ‘Convenient’ (26.85%), was characterized by the most frequent consumption of highly processed food products and water, a moderately frequent consumption of fruit, vegetables, and fruit and vegetables juices, and a low frequency consumption of meat and meat products, dairy products, and various other not recommended food products.

Pattern 2, ‘Non-meat’ (23.89%), was characterized by the lowest frequency of meat and meat products consumption, a high-frequency consumption of dairy products, different not recommended food products, and water, and a moderate consumption of highly processed food, vegetables and fruits, and vegetables and fruit juices.

Pattern 3, ‘Pro-healthy’ (24.38%), was characterized by the highest frequency of consumption of vegetables and fruits and vegetables and fruit juices, and the lowest frequency of consumption of highly processed food products. Consumption of the other foods was at a moderate frequency.

Pattern 4, ‘Carnivores’ (24.88%), was characterized by the highest frequency of consumption of meat and meat products, and the lowest intake of fruit and vegetables and water. Other foods (dairy products, vegetables and fruit juices, highly processed food products, and other not recommended food products) were consumed at a moderate level.

The final dataset consisted of 406 patients (382, 94.1% women; 24, 5.9% men; and women to men ratio of 15.9) with an average age of 35.6 years, the mean body mass of 70.1 kg, and BMI of 24.9. Most of the participants (54.2%) had a normal weight, were overweight (28.6%) or obese (11.3%), and only some were underweight (5.9%) (Table 3).

We found statistical differences between patterns regarding socio-demographic data (Table 3). The patients were mainly of the female gender (94.08%). They dominated the ‘Carnivores’ pattern (99.01%). The men were found the most in the ‘Non-meat’ pattern (11.34%). Participants were 32.37–37.67 years old. ‘Pro-healthy’ participants were the youngest on average, and ‘Non-meat’ participants were the oldest. However, we found differences in the type of work between patterns (*p* = 0.0397). The ‘Non-meat’ pattern had the lowest percentage of the unemployed, and the ‘Carnivores’ pattern had the highest (15.46% and 23.76%, respectively).

The brain-work type dominated in all patterns, and the ‘Non-meat’ pattern had the highest number of participants (73.2%), while the ‘Pro-healthy’ pattern had the smallest (54.55%). Other types of work like standing work and gardeners or farmers were observed in a small number of participants. We found that the ‘Pro-healthy’ pattern had more physical workers than other patterns.

All patterns were dominated by participants for which nutritional values and composition, and ecological origin of the food, bioproducts, and being on diet were the most important as a factor with the most substantial impact on food choice (Table 3). The highest proportion was found in ‘Carnivores’ (93.07%) and the least in ‘Pro-healthy’ (57.58%). The economical/financial aspect was the most important for ‘Pro-healthy’ (31.31%) and the least important for ‘Carnivores’ (4.95%). 

### 3.2. Dieting Experiences

The diet due to the diagnosis of thyroid disease in the last 2 years was followed by 61.5% of the respondents. Statistically, the highest percentage of respondents using this type of diet was found in ‘Non-meat’ (78.35%) and ‘Carnivores’ (71.29%) (*p* < 0.001) (Table 4). Adequately, the highest percentage of participants that had not been on any diet targeting thyroid disease in the last two years was in ‘Pro-healthy’ (52.53%), and the lowest was in ‘Non-meat’ (21.65%). 

At the time of research, 58.62% of respondents followed the diet for 3 months or longer. The differences between the patterns in terms of diet duration were statistically significant (*p* < 0.001). Correspondingly to the previous question, the highest number of participants that had not been on any diet were found in the pattern ‘Pro-healthy’ (58.59%), and the smallest proportion were found in the ‘Non-meat’ pattern (23.71%). 

Half of the respondents reported a much better well-being after the diet (any diet you have followed lately), and only 1.7% of the respondents felt worse (*p* < 0.0001). 

### 3.3. Disease and Health-Related Complaints Occurrence

Some diseases and ailments differentiated the patterns statistically significantly (Table 4). The highest percentage of respondents declared food allergies or food intolerances in the ‘Non-meat’ (13.4% or 32.99%, respectively) and “Carnivores’ (6.06% or 21.21%, respectively) patterns. Only 2.96% of the respondents reported having lipid diseases, with the largest number in the ‘Non-meat’ pattern (6.19%). Musculoskeletal ailments were reported by 59.85% of respondents, most of them in the ‘Pro-health’ pattern (72.73%).

### 3.4. Nutritional Behavior and Self-Assessment of the Nutrition

Patterns differed in most of the studied nutritional behaviors (Table 5). The identified patterns were statistically significantly different in terms of the regularity of meal consumption (*p* < 0.0001), eating breakfast before leaving the house (*p* = 0.0119), the frequency of eating between meals (*p* = 0.0242), and sweetening (*p* < 0.0001), as well as in the self-assessment of nutrition (*p* < 0.0001). Most of the respondents ate all or some meals regularly (79.06%). The highest percentage of those who consumed meals regularly was in the ‘Carnivores’ pattern (46.53%), and those who consumed some meals regularly in the ‘Convenient’ pattern (53.21%). Most subjects ate breakfast before leaving home (95.32%). The highest percentage of those who consumed breakfast before leaving home was in the ‘Non-meat’ pattern (58.76%), and those who consumed breakfast usually were most often found in the ‘Pro-health’ pattern (56.57%). Eating between meals was declared by 82.02% of respondents. People in the ‘Pro-health’ pattern most often declared eating between meals once/several times a day (55.56%). Conversely, eating between meals several times/1–2 times a week or never was reported by the majority of people in the ’Carnivores’ pattern (42.57% or 25.74%, respectively). 

Most of the respondents (76.85%) did not sweeten their foods or only did it occasionally, and most of them were in the ‘Convenient’ pattern (87.16%). Among those sweetening with 1–2 teaspoons of sugar/honey, the majority followed the ‘Pro-health’ pattern (33.33%). More than half of the respondents (68.97%) assessed their nutrition as good or very good. The largest number of such people followed the “Carnivores” pattern (88.12%), and the smallest number followed the “Pro-Health” pattern (36.36%).

### 3.5. Nutritional Knowledge

More than half of the respondents assessed their nutritional knowledge as good or very good (65.27%) (Table 5). The most people who assessed their nutritional knowledge as good were in the ‘Convenient’ pattern (46.79%), and very good in the ‘Carnivores’ pattern (32.67%).

### 3.6. Smoking Habits

Smoking cigarettes or tobacco statistically significantly differentiated the nutritional patterns (*p* = 0.0142) (Table 5). Most subjects were not smokers (89.66%), and they dominated the ‘Convenient’ pattern (96.33%). Most smokers were identified in the ‘Pro-health’ pattern (17.17%). 

### 3.7. Predictors of the Dietery Patterns

Data were adjusted for gender, BMI, and age. The results of logistic regression analysis are presented in Table 6.

People who did not eat breakfast before leaving home or who had food allergies and intolerances were less likely to follow the ‘Convenient’ pattern. In contrast, those who did not sweeten or sweetened only sometimes, or who did not smoke cigarettes or tobacco, were more likely to adhere to the ‘Convenient’ pattern. Respondents who ate some meals regularly or did not eat regularly and those who usually eat breakfast if they had time were less likely to adhere to the ‘Non-meat’ pattern. Brain workers, those who had food allergies or intolerances, lipid disorders, or had a good or very good nutrition self-assessment were more likely to adhere to the ‘Non-meat’ pattern. People for whom the most substantial impact on food choice was nutritional values and composition and ecological origin, etc., those who ate several times a week/1–2 times a week, or never/almost never between meals, or those who did not sweeten or sweetened sometimes, did not smoke cigarettes or tobacco, or self-assessed their nutrition as good or very good were less likely to adhere to the ‘Pro-health’ pattern. Physical workers, those who ate certain meals regularly or did not eat regularly, those who sometimes ate breakfast before leaving the house, or who did not eat breakfast were more likely to adhere to the ‘Pro-Health’ pattern. Respondents who ate some meals regularly or did not eat regularly were less likely to adhere to the ‘Carnivores’ pattern. People for whom the most substantial impact on food choice was nutritional values and composition and ecological origin, etc., those who ate several times a week/1–2 times a week or ate never/almost never between meals, those who did not sweeten or only sometimes sweetened their meals, those who had food allergies, and those who self-assessed their nutrition as good or very good were more likely to adhere to the ‘Carnivores’ pattern. 

## 4. Discussion

We assumed that the eating patterns of HT patients are intrinsically varied, and we hypothesized that healthy or unhealthy eating patterns differ depending on the socio-demographic characteristics and subjective well-being of HT patients. In line with our hypothesis, the eating patterns of patients with HT were internally differentiated. Healthy or unhealthy patterns differed markedly in the consumption of most types of food products and some characteristics, such as socio-demographic data, food choice motives, dietary experiences, and eating behavior. They also differed in the occurrence of certain diseases and ailments. The results of this study provide a better understanding of the eating behavior of people with an autoimmune disease. In this way, it becomes possible to explore options for better treatment of these patients based on the premise ‘if you know more, you can do more’.

### 4.1. Socio-Demographic Data

In our analysis, there were 382 females and 24 males that gave a gender ratio of 15.9. Compared to Omeljaniuk et al.’s results [21], our participants were on average 10 years younger, more of them had normal weight or were overweight, and less of them were obese. Unexpectedly, the highest proportion of females were found in the ’Carnivores’ pattern, while men were found mainly in the ’Non-meat’ pattern. Based on other studies [38] we could expect females to be found mainly in ’Pro-healthy’ pattern and men mainly in the ‘Carnivores’ pattern but we did not. Although, it should be mentioned that the number of men in the study was only 24 of 406 all participants. In other studies, the female to male ratio was at least 10:1 [39]. Although HT disease is dominant among women, it seems advisable to conduct similar studies in a well-chosen group of men, as the results may be interesting.

### 4.2. BMI, Obesity, and Dieting Experiences

Despite lack of statistical significance between patterns, BMI values showed that more than 40% of respondents should reduce body weight to a normal range. The proportion of participants with too high of a BMI score was lower than that found in the Polish National Health Program 2016–2020, where 57% of the 1017 representative participants of the population of Poland aged 18–86 years old had a BMI >25.0 kg/m^2^ [38]. Even small lifestyle changes like an increase in physical activity may improve thyroid functions [40]. Autoimmune hypothyroid diseases are related to increased concentrations of inflammatory and oxidative stress markers. Adipose tissue, especially visceral fat, is a source of adipokines and compounds that stimulate those processes [41]. On the other hand, the small group of respondents that was underweight in our study was also observed also in Omeljaniuk et al.’s study [21]. It confirms our clinical experience that hypothyroid patients do not have to be overweight or obese. Hypothyroidism and obesity are linked together because thyroid hormones regulate basal metabolism, thermogenesis, lipid and glucose metabolism, food intake, fat [40], and mitochondrial metabolism [42]. We know that HT patients also can lose weight and increasing body mass after hypothyroidism occurrence is not always the case. Low-grade chronic inflammation is associated with obesity [43] and might be a cause of decreased motivation of a patient to follow a diet or make dietary changes compared to non-obese patients [44]. The reason for that is the influence of inflammatory cytokines on the mesolimbic dopamine system, which is associated with reduced effort for the reward of dietary changes [43].

Polish women were found to have a higher concern about their health compared to men, which increased with age [44]. This suggests that there might be a potentially successful result of nutritional education of Polish women, especially if younger ones would get information about the long-term benefits of following healthy dietary patterns. Based on our clinical practice, we suggest that patients should be informed more about the potential health benefits of moving to healthier food choices than about the risks of inappropriate nutrition. Although the potential risks associated with excessive consumption of unhealthy foods are often underestimated [45], making the patient aware of them has certain benefits. Moreover, it was partly confirmed that Polish people were less likely to follow unhealthy dietary behaviors when they are more concerned about either health or nutrition [44]. One of the reasons why women had greater concern for nutrition might be that they perceive their image as overweight more than men [46]. Such perception might be a motivational factor for choosing healthier dietary patterns. In a study on the Polish population, women were less likely to adhere to unhealthy dietary patterns and had healthier dietary patterns than men [44]. Despite that, in our study, almost all participants were women, we did not find a lower number of subjects in unhealthy ‘Convenient’ dietary patterns or more participants in the ‘Pro-healthy” cluster.

In our study, many respondents had never dieted before. One of the reasons for that, despite their disease, might be chronic inflammation that decreases motivation for dietary changes. Another reason could be the low-quality services provided by medical specialists that recommend only hormone replacement therapy, thus disadvising or misadvising diet therapy. In our analysis, among those that were dieting, most of them did it for a time of no longer than 3 months, which is much too short to achieve desirable results for autoimmune disease. Some respondents were dieting for up to 12 months and longer and probably got better results than those who dieted for 3 months, if the diet was properly designed and followed. These results give us information that HT patients may have little or no experience with the diet. With an inflammatory-based disease, small dietary changes and easy-to-make recipes should be taken into account during planning dietary patterns. 

The degree of changes in nutrition should be taken individually. The short-term use of a diet in patients with autoimmune thyroid disease may be from the use of lactose-free and/ or gluten-free diets [19]. Both diets are very restrictive and difficult to follow. Most of those participants followed a diet without a dietitian’s support, basing it only on a medical recommendation. Following POLSPEN recommendations, gluten elimination is inappropriate for patients without gluten-related diseases [22].

From our practice, we were aware that HT patients follow different restrictive diets. We asked if they had been on any diet directed for thyroid disease in the past 2 years. By a thyroid disease diet, we meant any diet that they thought might improve their health. It was no surprise that among those following a diet that improved thyroid health, 67.2% had been on a gluten-free diet (GFD), lactose-free diet (LFD), or a combination of both. These diets are often recommended by medical specialists in Poland [19]. This result is consistent with the results of the Trofimiuk–Muldner study, in which 81.6% of patients with HT were on a gluten- or lactose-restricted diet [19]. Additionally, the Polish “LifeStyle Study” found that eating restrictions (e.g., calories, sugar, sweets, gluten, and fat restriction) are a common practice in over two-thirds of young women [47] and adult Poles [30]. We suppose that the percentage of participants restricting gluten or lactose in our study was higher than in the healthy Polish population. Young and adult Poles know the limits of the diet, and not only of these two nutrients [30,47]. However, we cannot compare our results with other studies. GFD and LFD are very restrictive and hard to follow. Even though gluten exclusion might be beneficial for HT patients [48], it is associated with potential exacerbation of nutritional deficiencies and carbohydrate or lipid metabolism in HT patients [18]. Lactose intolerance seems to occur more often in HT [49,50]; however, it should be implemented only when diagnosed based on the hydrogen and methane breath test [51]. In our study, 10.4% of patients used a vegetarian diet and 6% used the autoimmune protocol. We are going to test the effect of the autoimmune protocol in clinically diagnosed HT patients in another study. Surprisingly, only 2.4% of participants were on a thyroid-specific diet prescribed by a dietitian. 

Results of studies of Poles have shown that most of them undergo inappropriate nutrition habits [52]. Hashimoto’s patients from this country are not an exception and it may be concluded that they need dietary support because of malnutrition [21]. Unfortunately, a study done in 2019 found that most of them do not seek medical advice and even if they did, there is a high probability of getting an inappropriate dietary and supplementary treatment [19]. Nowadays, in our daily clinical practice, we see that hypothyroidism is treated mainly by endocrinologists pharmacologically through providing thyroid hormones; thus, nutritional intervention may differ from one specialist to another based on their knowledge [19]. We are still lacking recommendations about nutrition and supplementation in HT disease for both dietitians and doctors, on the basis of which they could lead the treatment [19]. 

### 4.3. Nutritional Patterns and Characteristic of Patterns

Respondents in the ‘Convenient’ pattern had the highest intake of ‘highly processed food products’ and the lowest of ’different not recommended’ foods (sweets, white rice, pasta, groats, white bread, and oils and margarines). There is a small chance that subjects following this pattern have a food allergy or food intolerance. We suppose that subjects from this pattern do not need to eliminate particular food products because of an allergy or intolerances [53] and thus choosing the convenient pattern of eating highly processed, ready to eat, or easy-to-make food products. Omeljaniuk et al. found, in a study of 101 Polish HT patients, that they consumed insufficient amounts of fat and dietary fiber and simultaneously had an excessive intake of processed food products high in easy-to-digest carbohydrates [21]. In another study, it was found that Polish people choose highly processed and lower-quality food products if they are financially poor [38]. The ‘Convenient’ pattern may relate to the way of living and concentrate on social meetings. We could suggest that is because highly processed food products like fast foods, energy drinks, sweetened drinks, and alcoholic drinks are consumed during social meetings. Other products from this pattern, such as canned meat and vegetables and powdered and ready-made soups, are easy and fast to make and eat. This is typical for students or may suggest that people following this pattern have a lack of time or minimal reflections about their health. In Borowiec and Aranowska’s study, the younger the woman participating in the study, the unhealthier their eating style was [38]. This suggests that, for younger people, the perspective of the disease seems to be less visible than for older people. Subjects in this pattern ate breakfast at home, most avoided salt use and sweetening, and were non-smokers. The majority considered their nutrition as good or very good, with self-assessed nutritional knowledge considered as good by half. This may be explained by the very low use of salt or sweeteners and smoking, which makes them believe they have a healthy diet and have adequate knowledge about nutrition. Another explanation might be that they compensate for the risk from eating low-nutrient highly processed food with avoidance of salt, sweeteners, and tobacco. Breakfast is also widely considered as the most important meal and that may be a reason for compensation risk also. Subjects in this pattern were almost free of lipid disorders but every second had musculoskeletal complaints. Musculoskeletal complaints are very often reported by HT patients due to dysfunction of mitochondrial oxidative phosphorylation [54]. We suggest that subjects in this pattern may suffer more often than those in other patterns because of the consumption of less nutritious food products [55]. Those nutrients might be magnesium, selenium, and iodine [56]. We cannot find an explanation for this pattern being free of lipid disorders as the consumption of highly processed food products is associated with cardiovascular diseases [56].

Subjects in the ‘Non-meat’ pattern had a higher chance of having a high intake of dairy products, different not recommended foods, and water, and a low intake of meat and meat products. There is a high probability that subjects in this pattern work mentally (office work), have food intolerances and allergies, and lipid disorders, as well as a good self-assessment of nutrition. Subjects who did not eat regularly and did not eat breakfast were more likely to adhere to this dietary pattern. It is surprising that ‘Non-meat’ subjects have a high intake of dairy products as those are often an issue for food allergies and intolerances [53]. The high intake of sweets, white rice, pasta, grouts, white bread, and oils and margarines may explain existence of lipid disorders in this pattern [56] despite the potentially beneficial effect of dairy products [57]. It is possible that the low nutritional value of different not recommended food products is one of factors responsible for the high occurrence of musculoskeletal complaints. An increase of plant-based food products probably would be found beneficial for this pattern for the reduction of musculoskeletal complaints [58]. The high proportion of subjects with different disease-related health issues might be an explanation for high attendance to diets targeting thyroid disease in the last two years. The feeling of improvement after dieting may motivate subjects to follow a diet. In fact, ‘Non-meat’ subjects were more likely stick to a diet of more than 12 months. It goes along with their self-assessment of nutrition as good or very good. However, the high intake of different not recommended food products stands opposite of this assessment. This dietary pattern might be improved along with the improvement of nutritional knowledge.

Subjects in the ‘Pro-heathy’ pattern ate or drank mainly vegetables and fruit, vegetables and fruit juice, water, and meat and meat products. There is a low chance that subjects consuming highly processed food products and different not recommended food products belong to this pattern. It could be suggested that a low intake of processed food is associated with compensation by vegetables and fruit and could be recommended to HT patients. Especially because HT patients, compared to control healthy people, had decreased glutathione levels even by up to 60% [59,60,61,62], and the level may increase with vegetable intake [63], and thus, decrease anti-TPO titers as they have been found to be correlated [62].

Subjects that choose food products based on their nutritional value had a low chance to belong to this pattern. There are discrepancies in this pattern as the incidence of particular food products indicates the ‘Pro-healthy’ pattern of nutrition, while nutritional behaviors differ from nutritional recommendations, which was unexpected. The data are self-reported; thus, they may be far from the truth. As we know, for example, in questionnaires regarding energy intake, participants tend to lie to protect their self-esteem [64]. It might also be true for answers regarding nutritional behaviors or smoking habits. This dietary pattern is also characterized by usually eating breakfast at home before leaving or not eating breakfast at all. This result was unexpected in regard to the social belief that breakfast is the most important meal. It might be a case that those subjects are aware of the positive effects of meal timing/intermittent fasting in which breakfast is often omitted [65]. Additionally, unexpectedly, non-smokers were more likely to belong to this pattern compared to smokers. 

Subjects in the ‘Carnivores’ pattern ate the most meat and meat products and drank vegetables and fruit juices rather than water. They ate vegetables and fruit and highly processed food to a lower extent. For subjects from this pattern nutritional values and composition of the food product is more important than the financial factor. This might be a protective factor against various diseases as processed red meat may increase the risk of the development of metabolic diseases, including obesity, cardiovascular diseases, and cancer [66,67]. Additionally, people having food allergies had higher chances of belonging to this pattern compared to subjects who did not have a food allergy. There is a high chance that people sometimes or never eating between meals, and those who do not or only rarely use sweeteners belong to this pattern. On the other hand, people who eat irregularly have a small chance of belonging to this pattern. They considered their way of eating as good.

Skipping breakfast seems to be a common practice in children, teenagers, and adults in Poland [68,69,70,71] and in Europe [72,73,74]. A high percentage of participants not eating breakfast at home or at all in our study was expected.

We found that dietary patterns detected in our study were similar to those found by other authors; however, they were not equal [7,11]. Thus, it was impossible to make a direct comparison between the results. It can be seen that the ‘Traditional Polish’ dietary pattern determined by Krusińska et al. [7] and Czarnocinska et al. [11] is similar to our ‘Convenient’ and ‘Carnivores’ patterns, because of the consumption of processed food products and meat. In our study, subjects in the ‘Pro-healthy’ pattern had a high-frequency consumption of fruit and vegetables similar to those in the ‘Prudent’ [7] or ‘Fruit and vegetables’ dietary patterns [11] in studies of other authors. In our study, we included a ‘Non-meat’ pattern in which consumption of meat and meat products was the lowest among all the patterns. No such data were found in the above-mentioned studies. We suggest that unhealthy food products, including in the ‘Convenient’” pattern, should be limited and patients should be advised towards a higher consumption of fruit and vegetables, similar to the ‘Pro-health’ pattern. Nevertheless, the restrictions of particular food groups can be a predictor of both healthy and unhealthy dietary patterns, at least in the population of young Polish girls [45]. As dietary patterns in different regions of Poland are differentiated, we suggest that they are internally different in HT patients, as well as between those with different regional origins [29,30,31].

### 4.4. Strenghts, Limitations, and Future Perspective

The strength of our study is a relatively large sample of Polish HT patients who were from different regions of the country. It is also the first study to identify the eating patterns of Polish HT patients. We also used a questionnaire developed based on the validated KomPAN^®^ food frequency questionnaire [32,33].

The main limitation of our study is the use of a questionnaire in which potential biases may occur when self-reported data is analyzed, e.g., subjects may overestimate the consumption of some foods [75] and/or underreport the consumption of food products considered as unhealthy (e.g., sweets) [76]. The second limitation is the inability to quantify food or nutrient intake. We also were not able to collect biochemical measurements as it was logistically impossible. In future studies, biochemical tests would either let researchers exclude self-diagnosed subjects or study only hypothyroid ones. Our results may be related only to Polish subjects as nutritional patterns or behaviors are based on many factors like cultural background and other components [29,30,31]. Moreover, they differ in individual regions of Poland [11,77]; thus, it seems reasonable to study subjects from particular regions of Poland or to divide them based on their sedentism and/or origin. For example, the Northern region seems to consume more dairy and fats, which suggests the influence of Baltic and Scandinavian countries, while differences between Southern and Western regions might be explained by strong influence in the past by Russian and German cultures. There are other components that worth be taking into consideration, e.g., the ‘Traditional Polish’ pattern among young girls may be more related to their socioeconomic status rather than being a region-specific pattern [11]. Unfortunately, we do not know how dietary patterns changes in subjects after they are diagnosed. Drywień et al. found also that nutritional patterns change with age from teenagers to older Polish individuals [78]. In our study, we did not divide subjects based on their age. This could be taken into consideration in future studies, as age and BMI are considered as potential confounders [77]. In our study, we aimed to identify nutritional patterns particularly in HT patients. Other limitations are the lack of a control group and no established causal relationship between the studied variables, but these may be guidelines for further studies.

### 4.5. From the Point of View of a Clinical Dietitian Working with HT Patients

At the time of publishing this manuscript, there was only one qualitative dietary protocol (Diet4Hashi) proposed by Wojtas et al. [27] in the literature, which was developed through work with subjects suffering from HT. We consider the above-mentioned protocol as potentially valuable and easy to use by patients. For better clinical outcomes in patient management, more attention should be paid to the factors outlined below. Most importantly, a medical specialist who is not familiar with clinical nutrition should advise his patient to work with a clinical dietitian. Endocrinologists could also give such a protocol to their patients so that they do not have to search for it in non-medical, unscientific sources, as it is now common practice [19].

The simplified advice ‘eat more vegetables and fruits’ may not be followed because the patient does not know what is actually meant by this guideline. As mentioned in the article, a healthy diet based on a higher frequency of plant consumption is beneficial to the entire population, both healthy and sick people, including those with HT. If the transition is for a patient on a typical Western diet, better outcomes could be expected. We suggest that understanding the importance of a patient’s changing eating behavior and eating patterns would increase the likelihood of their implementation. This may increase the chances of developing good eating habits and result in better clinical outcomes of therapy. Therefore, we propose for specialists that they introduce their patients to the concepts of oxidative stress and inflammatory processes, their relations with each other, their relations with HT, and the influence of antioxidants on the anti-inflammatory potential of human serum.

To our knowledge, there is no specific diet that has been studied in HT patients. We know of some studies that test an ‘autoimmune protocol’ that aims to reduce autoimmune processes. However, no final dietary pattern has been established and more research is needed. Despite the potential benefits found in, e.g., Abbott et al. [79], studies were conducted with multidisciplinary changes in diet and/or lifestyle, so it is difficult to identify specific causes of the changes. These types of results vary depending on the study and the authors’ approach. Therefore, there is no representative pattern to follow. There is also no unambiguous conclusion as to which change or to what extent that change was responsible for the positive results.

We suggest that professionals inform patients about the importance of nutrients, such as selenium, vitamin D [80] and others [5], in their treatment and in which foods they can be found. In the Polish population, including HT patients, the excessive consumption of protein, fat, and energy-dense foods, as well as the deficiency of polyunsaturated fatty acids and fiber, are worrying [21]. The Polish population is often malnourished and depleted in nutrients, such as selenium, vitamin D [80], magnesium [81,82], and B vitamins [81], so a complete analysis of patients’ eating patterns and blood tests should be performed to obtain detailed knowledge. Tests should also include other nutrients important to the health of the thyroid gland, such as iron (ferritin to assess iron metabolism), zinc, and lipophilic vitamins.

We also met many patients who were potentially iodine deficient or who were supplementing with iodine in dangerously high daily doses. The patient should know the importance of iodine for thyroid function, but also the danger of its deficiency and excess [5]. Iodine deficiency, in addition to other nutrients, may result from dietary restrictions, e.g., as a result of a vegan or vegetarian diet [83]. In Poland, as in other countries, iodine deficiency continues to be a problem [84]. Since there is no diet that fully covers iodine requirements [84], patients should be advised to consume foods containing iodine.

Unfortunately, some patients still remain misdiagnosed or undiagnosed. However, this is not the subject of the manuscript, so interested readers are referred to the literature for guidance in the diagnosis of NT disease. In short, it is inappropriate to make a diagnosis based on serum TSH levels alone. A clinical dietitian, especially an endocrinologist, should know about the sick euthyroid syndrome (SES) [85,86] when working with a patient.

## 5. Conclusions

Hashimoto’s patients differ in their eating behavior and dietary experiences and thus represent different eating patterns. Unexpectedly, we did not find more participants in healthier dietary patterns, e.g., with a higher frequency of consumption of fruit and vegetables. However, these patterns are not determined by comorbidities or ailments. Food allergies and intolerances were the only determinants, but these did not lead HT patients to follow the pro-healthy formula. Therefore, it may be assumed that, for example, eating habits, lifestyle, or socio-demographic conditions other than those examined here may play a stronger role in maintaining certain dietary patterns in patients with HT. Future research should focus on finding other determinants of food choice and nutritional behavior in HT patients in particular regions of Poland. Perhaps the identified dissonance between the type of food chosen and eating behavior may have a psychological basis and here the sources should be looked for.

## Figures and Tables

**Table 1:** Factor-loading matrix identified by factor analysis.

Product	Highly Processed Food Products	Dairy Products	Meat and Meat Products	Different Not Advised	Vegetables and Fruits	Vegetables and Fruit Juices	Water
Powder soups	0.780	-	-	-	-	-	-
Canned meat	0.752	-	-	-	-	-	-
Ready-made soups (e.g., in cardboard packages)	0.711	-	-	-	-	-	-
Fast food	0.678	-	-	-	-	-	-
Energy drinks	0.675	-	-	-	-	-	-
Alcoholic drinks	0.574	-	-	-	-	-	-
Sweetened drinks	0.546	-	-	-	-	-	-
Canned and marinated vegetables	0.526	-	-	-	-	-	-
Yogurt, kefir	-	0.825	-	-	-	-	-
Cottage cheese	-	0.824	-	-	-	-	-
Milk	-	0.722	-	-	-	-	-
Yellow cheeses	-	0.649	-	-	-	-	-
Wholegrain bread	-	0.469	-	-	-	-	-
Butter	-	0.452	-	-	-	-	-
Homogenized cheese	-	0.428	-	-	-	-	-
Red meat dishes	-	-	0.791	-	-	-	-
White meat dishes (poultry, rabbit)	-	-	0.722	-	-	-	-
Sausages or frankfurters	-	-	0.721	-	-	-	-
Fried foods (e.g., meat or flour based)	-	-	0.486	-	-	-	-
Fish	-	-	0.430	-	-	-	-
Lard	-	-	0.410	-	-	-	-
Sweets	-	-	-	0.566	-	-	-
White rice, pasta, groats	-	-	-	0.542	-	-	-
White bread	-	-	-	0.529	-	-	-
Oils and margarines	-	-	-	0.419	-	-	-
Vegetables	-	-	-	-	0.736	-	-
Fruits	-	-	-	-	0.700	-	-
Legume-based meals	-	-	-	-	0.600	-	-
Buckwheat, oats, wholegrain pasta	-	-	-	-	0.475	-	-
Fruit juices	-	-	-	-	-	0.765	-
Vegetables or vegetable and fruit juices	-	-	-	-	-	0.755	-
Water, e.g., mineral and table water	-	-	-	-	-	-	0.602
Variance explained (%)	22.35%	8.06%	7.27%	5.89%	4.52%	3.72%	3.27%
Total Variance explained (%)	55.08%						
Cronbach’ alpha	0.842	0.804	0.734	0.696	0.682	0.811	-
Kaiser’s Measure of Sampling Adequacy:	0.862						

**Table 2:** Characteristics of the identified patterns according to the consumption frequency; the mean ratings of the patterns on the classification variables.

	Dietary Patterns				
Variables	Convenient Pattern 1 *n* = 109	Non-Meat Pattern 2 *n* = 97	Pro-Healthy Pattern 3 *n* = 99	Carnivores Pattern 4 *n* = 101	*p*-Value
F1: Highly processed food products	4.39 ^a,^*	2.98 ^b^	1.81 ^d^	2.66 ^c^	<0.0001
F2: Dairy products	2.42 ^d^	3.62 ^a^	2.77 ^c^	3.24 ^b^	<0.0001
F3: Meat and meat products	2.59 ^c^	1.63 ^d^	3.59 ^b^	4.15 ^a^	<0.0001
F4: Different not recommended products	1.88 ^d^	3.98 ^a^	2.64 ^c^	3.57 ^b^	<0.0001
F5: Vegetables and fruit	2.63 ^b^	2.88 ^b^	4.54 ^a^	1.97 ^c^	<0.0001
F6: Vegetables and fruit juices	3.18 ^a^	2.57 ^b^	3.12 ^a^	3.06 ^a^	0.0096
F7: Water	3.49 ^a^	3.37 ^a^	2.97 ^b^	2.10 ^c^	<0.0001

*, a, b, c, d—Means with the same letter are not significantly different; ANOVA post-hoc Waller–Duncan K-ratio *t* Test.

**Table 3:** Characteristics of respondents by dietary patterns (%).

		Dietary Patterns					
	Variables	Convenient *n* = 109	Non-Meat *n* = 97	Pro-Healthy *n* = 99	Carnivores *n* = 101	*p*-Value	Total (*n*)
Age	-	35.04 ^b^*	36.67 ^a^	32.37 ^c^	33.26 ^bc^		
Gender	Female	94.5	88.66	93.94	99.01	0.0226	382
	Male	5.5	11.34	6.06	0.99		24
BMI categories	Underweight	5.5	6.19	4.04	7.92	0.7069	24
	Normal weight	56.88	50.52	49.49	59.41	-	220
	Overweight	24.77	31.96	34.34	23.76	-	116
	Obese	12.84	11.34	12.12	8.91	-	46
Type of work	Unemployed	22.94	15.46	18.18	23.76	0.0397	82
	Brain work	60.55	73.2	54.55	56.44		248
	Physical work	9.17	5.15	14.14	4.95		34
	Standing work	7.34	6.19	13.13	14.85		43
Factors with the most substantial impact on food choice	Economical/ financial	21.1	14.43	31.31	4.95	< 0.0001	73
	Nutritional values and composition, and ecological origin of the food, bioproducts, and being on a diet	74.31	83.51	57.58	93.07		313
	Advertisement of a particular food product, a fad for its consumption, gustatory preferences	2.75	1.03	4.04	0.0		8
	Time/time of meal preparation/food products’ availability	0.00	0.00	5.05	1.98		7
	I do not make shopping trips/ I do shopping randomly	1.83	1.03	2.02	0.0		5

*, a, b, c—Means with the same letter are not significantly different in the ANOVA test with post hoc Waller–Duncan K-ratio *t*-test. Red color—values are statistically significant.

**Table 4:** Characteristics of the respondents by diseases, well-being, diet, and dietary patterns (%).

		Convenient *n* = 109	Non-Meat *n* = 97	Pro-Healthy *n* = 99	Carnivores *n* = 101	*p*-Value	Total (*n*)
Diet targeting thyroid disease in the last two years	Yes	50.46	78.35	47.47	71.29	<0.001	250
	No	49.54	21.65	52.53	28.71		156
The current duration of the diet	No diet	49.54	23.71	58.59	32.67	<0.001	168
	Up to 3 months	22.02	20.62	21.21	30.69		96
	4–12 months	20.18	19.59	9.09	18.81		69
	More than 12 months	8.26	36.08	11.11	17.82		73
Well-being after the diet (any diet you have followed lately)	Much better	74.07	83.78	84.78	87.32	<0.0001	203
	Hard to assess	20.37	13.51	10.87	12.68		35
	Worse	5.56	2.7	4.35	0.00		7
Having diabetes	No	97.25	95.88	96.97	98.02	0.8461	394
	Yes	2.75	4.12	3.03	1.98		12
Having any cardiovascular disease	No	95.41	94.85	96.97	96.04	0.8935	389
	Yes	4.59	5.15	3.03	3.96		17
Having food allergies	No	96.33	86.6	93.94	86.14	0.0193	369
	Yes	3.67	13.4	6.06	13.86		37
Having food intolerances	No	83.49	67.01	78.79	73.27	0.0377	308
	Yes	16.51	32.99	21.21	26.73		98
Having any intestinal disease	No	87.16	85.57	88.89	83.17	0.6825	350
	Yes	12.84	14.43	11.11	16.83		56
Having kidney failure	No	100	100	100	100	-	406
	Yes	0	0	0	0		0
Having gout	No	100	100	100	100	-	406
	Yes	0	0	0	0		0
Having lipid disorders	No	99.08	93.81	94.95	100	0.0213	394
	Yes	0.92	6.19	5.05	0		12
Gastrointestinal complaints *	No	27.52	22.68	13.13	25.74	0.0661	91
	Yes	72.48	77.32	86.87	74.26		315
Nervous system complaints *	No	22.02	16.49	10.1	12.87	0.0962	63
	Yes	77.98	83.51	89.90	87.13		343
Musculoskeletal complaints *	No	50.46	37.11	27.27	44.55	0.005	163
	Yes	49.54	62.89	72.73	55.45		243
Skin complaints *	No	17.43	27.84	21.21	22.77	0.3481	90
	Yes	82.57	72.16	78.79	77.23		316

* Gastrointestinal complaints (including heartburn, constipation, diarrhea, vomit, taste disturbance, stomachache, and bloatedness); nervous system complaints (concentration problems, fatigue, sleepiness, depressive states, and headache); musculoskeletal complaints (muscle cramps, arthralgia, edema, and swelling); skin complaints (hair loss, brittle nails, dry skin, and skin problems). Red color—value is statistically significant.

**Table 5:** Characteristics of the respondents by nutritional behavior, nutritional knowledge, smoking, and dietary patterns (%).

		Convenient	Non-Meat	Pro-Healthy	Carnivores	*p*-Value	Total (*n*)
Number of meals during the day	1–2	0.92	5.15	5.05	0.99	0.1883	-
	3–4	81.65	72.16	78.79	72.25		313
	≥5	17.43	22.68	16.16	23.76		-
Regularity of meals	Yes	28.44	44.33	14.14	46.53	<0.0001	135
	Yes, some meals	53.21	38.14	48.48	42.57		186
	No	18.35	17.53	37.37	10.89		85
Eating breakfast before leaving the house	Yes, always	55.96	58.76	36.36	53.47	0.0119	208
	Usually	43.12	35.05	56.57	41.58		179
	No	0.92	6.19	7.07	4.95		19
The frequency of eating between meals	Once/several times a day	45.54	43.3	55.56	31.68	0.0242	183
	Several times a week/1–2 times a week	35.78	37.11	32.32	42.57		150
	Never/almost never	14.68	19.59	12.12	25.74		73
Salting	Yes, for most meals	13.76	15.46	18.18	11.88	0.7495	60
	Yes, sometimes	32.11	30.93	37.37	35.64		138
	No, I do not	54.13	53.61	44.44	52.48		208
Sweetening	Yes, 1–2 teaspoons of sugar/honey	11	16.5	33.33	8.91	<0.0001	70
	Yes, I use sweeteners	1.84	8.24	8.08	5.94		24
	No, I do not or only sometimes	87.16	75.26	58.59	85.15		312
Self-assessment of nutrition	Bad or very bad	25.69	23.71	63.64	11.88	<0.0001	126
	Good or very good	74.31	76.29	36.36	88.12		280
Self-assessment of nutrition during the week compared to the weekends	No different	47.71	52.58	44.44	51.49	0.5358	199
	Differs slightly	37.61	39.18	43.43	41.58		164
	Varies greatly	14.68	8.25	12.12	6.93		43
Self-assessment of nutritional knowledge	Inadequate	11.01	8.25	23.23	4.96	0.0002	48
	Adequate	24.77	17.53	28.28	20.79		93
	Good	46.79	45.36	35.35	41.58		172
	Very good	17.43	28.87	13.13	32.67		93
Smoking cigarettes or tobacco	Yes	3.67	9.28	17.17	11.88	0.0142	42
	No	96.33	90.72	82.83	88.12		364

Red color—value is statistically significant.

**Table 6:** Associations between dietary patterns and selected characteristics of the study sample (odds ratios).

Variables	Convenient		Non-Meat		Pro-Healthy		Carnivores	
	* OR (95% CI) *p*	aOR (95% CI) *p*	OR (95% CI) *p*	aOR (95% CI) *p*	OR (95% CI) *p*	aOR (95% CI) *p*	OR (95% CI) *p*	aOR (95% CI) *p*
Type of work:								
Unemployed	1	1	1	1	1	1	1	1
Office work	0.83 (0.48; 1.43) 0.4964	0.80 (0.46; 1.39) 0.4351	1.79 (1.06; 3.34) 0.0469	1.88 (1.03; 3.55) 0.0418	0.99 (0.54; 1.81) 0.9732	1.03 (0.56; 1.89) 0.9295	0.72 (0.41; 1.26) 0.2528	0.69 (0.39; 1.22) 0.2044
Physical work	0.95 (0.4; 2.28) 0.9085	0.93 (0.38; 2.24) 0.8663	0.77 (0.26; 2.32) 0.6422	0.72 (0.23; 2.22) 0.567	2.48 (1.05; 5.88) 0.0377	2.50 (1.05; 5.98) 0.0389	0.42 (0.14; 1.21) 0.106	0.45 (0.15; 1.31) 0.1431
Standing work	0.54 (0.22; 1.32) 0.1762	0.49 (0.2; 1.22) 0.1249	0.74 (0.27; 2.09) 0.5743	0.84 (0.29; 2.39) 0.743	1.59 (0.69; 3.68) 0.2753	1.75 (0.74; 4.1) 0.2001	1.34 (0.61; 2.96) 0.465	1.22 (0.54; 2.73) 0.6332
Factors with the most substantial impact on food choice:								
Economical/financial	1	1	1	1	1	1	1	1
Nutritional values and composition, and ecological origin of the food, bioproducts, and being on a diet	0.76 (0.44; 1.32) 0.33	0.75 (0.43; 1.32) 0.3147	1.47 (0.78; 2.78) 0.2334	1.49 (0.78; 2.85) 0.2317	0.30 (0.18; 0.52) <0.0001	0.30 (0.17; 0.53) <0.0001	5.84 (2.28; 14.94) <0.0002	5.96 (2.31; 15.37) <0.0002
Number of meals during the day:								
1–2	1	1	1	1	1	1	1	1
3–4	4.37 (0.56; 34.35) 0.1609	4.42 (0.56; 34.86) 0.1589	0.40 (0.12; 1.31) 0.1308	0.44 (0.13; 1.47) 0.1812	0.47 (0.14; 1.51) 0.2014	0.45 (0.14; 1.48) 0.1883	3.52 (0.45; 27.7) 0.2314	3.18 (0.4; 25.48) 0.2765
≥5	3.37 (0.41; 27.82) 0.2591	3.48 (0.42; 28.9) 0.2485	0.52 (0.15; 1.82) 0.3072	0.59 (0.16; 2.1) 0.4134	0.35 (0.1; 1.23) 0.1005	0.32 (0.09; 1.18) 0.0866	4.63 (0.57; 37.8) 0.153	4.56 (0.49; 33.81) 0.1956
Regularity of meals:								
Yes	1	1	1	1	1	1	1	1
Yes, some meals	1.52 (0.92; 2.52) 0.1055	1.53 (0.92; 2.55) 0.1031	0.53 (0.32; 0.89) 0.0152	0.52 (0.31; 0.88) 0.0137	3.01 (1.58; 5.72) 0.0008	3.02 (1.58; 5.77) 0.0008	0.56 (0.34; 0.92) 0.022	0.56 (0.34; 0.92) 0.0221
No	1.03 (0.54; 1.96) 0.9228	1.06 (0.55; 2.02) 0.8676	0.54 (0.28; 0.96) 0.0465	0.54 (0.28; 0.94) 0.0437	6.66 (3.31; 13.41) <0.0001	6.52 (3.21; 13.23) <0.0001	0.28 (0.14; 0.58) 0.0006	0.27 (0.13; 0.57) 0.0005
Eating breakfast before leaving the house:								
Yes, always	1	1	1	1	1	1	1	1
I usually do if I have time	0.86 (0.55; 1.34) 0.5022	0.84 (0.53; 1.32) 0.4482	0.62 (0.38; 0.98) 0.0429	0.60 (0.37; 0.99) 0.0448	2.18 (1.35; 3.51) 0.0015	2.24 (1.38; 3.65) 0.0012	0.87 (0.55; 1.39) 0.5707	0.86 (0.55; 1.42) 0.614
I do not eat breakfast	0.13 (0.02; 0.93) 0.0329	0.13 (0.02; 0.97) 0.0463	1.22 (0.44; 3.37) 0.6973	1.18 (0.42; 3.32) 0.7536	2.79 (1.03; 7.57) 0.0443	2.93 (1.06; 8.05) 0.0376	1.02 (0.35; 2.96) 0.9731	1.11 (0.37; 3.30) 0.8517
The frequency of eating between meals:								
Once/several times a day	1	1	1	1	1	1	1	1
Several times a week/1–2 times a week	0.84 (0.52; 1.36) 0.478	0.82 (0.50; 1.34) 0.4304	1.06 0,8221 0.8221	1.07 (0.64; 1.81) 0.7924	0.63 (0.38; 0.94) 0.0426	0.64 (0.39; 0.97) 0.0417	1.89 (1.13; 3.19) 0.0159	1.90 (1.12; 3.24) 0.0182
Never/almost never	0.67 (0.35; 1.27) 0.2204	0.65 (0.34; 1.26) 0.2008	1.18 (0.63; 2.21) 0.6021	1.08 (0.56; 2.09) 0.8211	0.46 (0.23; 0.92) 0.0276	0.47 (0.23; 0.95) 0.0367	2.61 (1.42; 4.82) 0.0021	2.96 (1.56; 5.63) 0.0009
Salting:								
Never/almost never	1	1	1	1	1	1	1	1
Yes, for most meals	1.02 (0.51; 2.05) 0.957	1.01 (0.50; 2.04) 0.9877	0.83 (0.41; 1.7) 0.6151	0.86 (0.42; 1.77) 0.6807	0.86 (0.44; 1.67) 0.6453	0.91 (0.46; 1.79) 0.7857	1.41 (0.68; 2.95) 0.3599	1.31 (0.62; 2.77) 0.4834
No, I do not	1.19 (0.62; 2.29) 0.6077	1.17 (0.6; 2.28) 0.6363	1 (0.52; 1.94) 1	1.04 (0.53; 2.04) 0.9195	0.63 (0.33; 1.19) 0.1544	0.66 (0.35; 1.28) 0.2186	1.37 (0.68; 2.77) 0.3843	1.27 (0.62; 2.60) 0.5154
Sweetening:								
Yes, 1–2 teaspons of sugar/honey	1	1	1	1	1	1	1	1
Yes, I use sweeteners	0.44 (0.09; 2.12) 0.3062	0.44 (0.09; 2.12) 0.3022	1.69 (0.61; 4.66) 0.3122	1.65 (0.59; 4.66) 0.3435	0.56 (0.21; 1.48) 0.2421	0.56 (0.21; 1.50) 0.2512	2.26 (0.71; 7.20) 0.1682	2.35 (0.72; 7.69) 0.1573
No, I do not or only sometimes	2.12 (1.09; 4.12) 0.0276	2.13 (1.09; 4.18) 0.0274	1.03 (0.56; 1.91) 0.923	1.07 (0.57; 2.01) 0.8336	0.26 (0.15; 0.44) <0.0001	0.26 (0.15; 0.45) <0.0001	2.58 (1.23; 5.42) 0.0124	2.46 (1.16; 5.22) 0.0186
Having diabetes:								
No	1	1	1	1	1	1	1	1
Yes	0.91 (0.24; 3.41) 0.8835	1.01 (0.26; 3.86) 0.9943	1.62 (0.48; 5.50) 0.4398	1.36 (0.38; 4.87) 0.9943	1.04 (0.28; 3.90) 0.9595	0.91 (0.24; 3.50) 0.888	0.59 (0.13; 2.77) 0.5087	0.73 (0.15; 3.49) 0.6881
Having any cardiovascular disease:								
No	1	1	1	1	1	1	1	1
Yes	1.14 (0.39; 3.32) 0.8075	1.14 (0.39; 3.36) 0.8135	1.35 (0.46; 3.92) 0.5867	1.25 (0.42; 3.74) 0.6904	0.65 (0.18; 2.33) 0.5121	0.61 (0.17; 2.20) 0.4495	0.93 (0.30; 2.91) 0.8956	1.09 (0.34; 3.55) 0.882
Having food allergies:								
No	1	1	1	1	1	1	1	1
Yes	0.31 (0.11; 0.88) 0.0283	0.28 (0.10; 0.82) 0.0203	1.84 (0.90; 3.77) 0.0964	2.05 (1.09; 4.25) 0.0447	0.57 (0.23; 1.42) 0.23	0.59 (0.24; 1.46) 0.2505	1.97 (1.07; 4.00) 0.0394	1.91 (1.03; 3.92) 0.0479
Having food intolerances:								
No	1	1	1	1	1	1	1	1
Yes	0.54 (0.30; 0.95) 0.0313	0.53 (0.30; 0.93) 0.0281	1.81 (1.1; 3.00) 0.0205	1.92 (1.15; 3.21) 0.0124	0.80 (0.47; 1.39) 0.4346	0.83 (0.48; 1.44) 0.507	1.20 (0.72; 2.01) 0.4823	1.13 (0.67; 1.91) 0.6374
Having any intestinal disease:								
No	1	1	1	1	1	1	1	1
Yes	0.89 (0.47; 1.71) 0.7374	0.88 (0.46; 1.69) 0.7039	1.07 (0.56; 2.06) 0.8341	1.07 (0.55; 2.08) 0.8345	0.73 (0.36; 1.47) 0.3752	0.75 (0.37; 1.51) 0.4153	1.38 (0.74; 2.57) 0.3083	1.38 (0.73; 2.58) 0.3234
Having lipid disorders:								
No	1	1	1	1	1	1	1	1
Yes	0.24 (0.03; 1.89) 0.1753	0.23 (0.03; 1.85) 0.1673	3.33 (1.05; 10.58) 0.0413	3.38 (1.04; 11.05) 0.0436	2.28 (0.71; 7.35) 0.1678	2.09 (0.64; 6.86) 0.2244	-	-
Smoking cigarettes or tobacco:								
Yes	1	1	1	1	1	1	1	1
No	3.85 (1.34; 11.06) 0.0122	3.91 (1.35; 11.32) 0.0124	1.17 (0.54; 2.54) 0.6928	1.10 (0.5; 2.43) 0.8068	0.43 (0.22; 0.83) 0.0121	0.43 (0.22; 0.85) 0.0152	0.81 (0.4; 1.65) 0.5591	0.83 (0.4; 1.71) 0.608
Self-assessment of nutrition:								
Bad or very bad	1	1	1	1	1	1	1	1
Good or very good	1.43 (0.87; 2.33) 0.1596	1.49 (0.89; 2.52) 0.1309	1.61 (0.95; 2.72) 0.0757	1.69 (1.04; 2.96) 0.0423	0.15 (0.09; 0.24) <0.0001	0.13 (0.08; 0.22) <0.0001	4.43 (2.32; 8.44) <0.0001	4.81 (2.43; 9.51) <0.0001

* OR—odds ratio, aOR—adjusted odds ratio. Red color—variable is statistically significant (*p* < 0.05).

## Data Availability

The data presented in this study are available on request from the corresponding author.
